# The non-haemorrhagic vagal response to trauma: a review of hypotensive and bradycardic responses to injury in the absence of bleeding

**DOI:** 10.1007/s00068-024-02648-y

**Published:** 2024-09-04

**Authors:** Jonathan Woods, Jake Turner, Amy Hughes, Gareth Davies, Gareth Grier

**Affiliations:** 1 Barts and The London School of Medicine and Dentistry, Queen Mary University of London, Mile End Road, London, E1 4NS England; 2 Nottingham University Hospitals NHS Trust, Hucknall Road, Nottingham, NG5 1PB Nottinghamshire England; 3 Emergency Department, Manx Care, Nobles Hospital, Braddan, IM4 4RJ Isle of Man

**Keywords:** Trauma, Vagus nerve, Vital signs, Haemorrhage

## Abstract

**Purpose:**

Trauma has the potential to cause haemorrhage, tissue damage, pain, visceral manipulation and psychological distress. Each of these consequences of trauma can cause changes in autonomic outflow, which dictates a patient’s vital signs. Patients who are hypotensive and bradycardic due to a vagally mediated parasympathetic response to pain, psychological distress and visceral manipulation may be confused with those who exhibit bradycardia and hypotension following significant blood volume loss.

**Methods:**

This review summarises literature that describes specific stimuli, patterns of injury and patient characteristics that are associated with a non-haemorrhagic vagal response to trauma.

**Results:**

Twenty-six records described predominantly parasympathetic responses to trauma (both blunt and penetrating) and surgery (“iatrogenic trauma”). Such a non-haemorrhagic vagal response occurs following a wide variety of injury patterns. Patient age and sex are poor predictors of the likelihood of a non-haemorrhagic vagal response. The development and resolution of a non-haemorrhagic vagal response occurs over a heterogenous time period. It is unclear whether speed of onset and resolution is linked to the pattern of injury or other factors causing a predominantly parasympathetic response following non-haemorrhagic trauma.

**Conclusion:**

The pattern of injury, patient demographic and speed of onset / resolution associated with the non-haemorrhagic vagal response to trauma may is heterogenous. It is therefore challenging to clinically distinguish between the hypotensive bradycardia due to hypovolaemia secondary to haemorrhage, or a parasympathetic response to trauma in the absence of bleeding.

**Supplementary Information:**

The online version contains supplementary material available at 10.1007/s00068-024-02648-y.

## Background

Patients who suffer traumatic injuries, undergo surgery (“iatrogenic trauma”) or even non-invasive procedures may experience stimuli that cause haemorrhage, tissue damage, pain, visceral manipulation and psychological distress to varying extents. Each of these consequences of trauma has a different impact on the patient’s physiology, mediated in part by the autonomic nervous system, and measured using vital signs such as heart rate and blood pressure.

### Haemorrhage

The Advanced Trauma Life Support (ATLS) classification of haemorrhagic shock suggests that, with up to 30% of blood loss, a patient becomes tachycardic in order to maintain their blood pressure. Beyond 30% of blood loss, the tachycardia increases, but is unable to compensate for the lost volume and the patient becomes hypotensive [1]. However, data from as early as the First World War that describes an absence of tachycardia in cases of severe blood loss [2], brings this model into question. More recent clinical studies [3, 4] have concluded that the interrelations between clinical signs such as heart rate and blood pressure are not seen “to the same degree as that suggested by the ATLS classification of shock” [4]. Little et al.’s review [5] of blood pressure and heart rate during venesection (blood loss without concurrent traumatic tissue damage) describes a biphasic response to hypovolaemia. Initially, healthy volunteers mounted a tachycardia [6, 7], mediated by the baroreceptor response in order to maintain blood pressure [5]. After 15% of blood was lost, the subjects experienced profound hypotension and bradycardia before losing consciousness. This was thought to be due to incomplete filling of the heart causing cardiac vagal C-fibres [8] in the left ventricular myocardium to initiate the “cardiac reflex”: increased vagal outflow to the heart and decreased sympathetic tone in peripheral vasculature. There has been little modern research into the basic physiological reflexes and interaction between them in the human response to trauma. Studies of trauma patients have noted that many patients who have mechanisms of injury compatible with haemorrhage can present without tachycardia [9–13]. Observational studies of large cohorts of trauma patients by Victorino [9] and Ley [10] show that the incidence of “relative bradycardia”^1^is as high as 35% or 44% respectively. Sander-Jensen’s [12] case series of hypovolaemic patients showed the heart rate can fall to as low as 46 beats per minute (bpm) despite hypotension. Furthermore, Demetriades’ study [13] of 750 hypotensive trauma patients found that those presenting with bradycardia had a better, but less predictable prognosis (bradycardia group crude mortality = 21.7%, tachycardia group = 29.2%, p = 0.047). This finding challenges the previously proposed hypothesis [14] that bradycardia represents a preterminal state of hypovolaemia. However, it also raises the possibility that hypotensive bradycardia in trauma may not always be caused by isolated severe hypovolaemia. These haemodynamic changes may be mediated by other pathways triggered by the psychological and physiological consequences of trauma. Such consequences may not cause life threatening haemorrhage but may cause a patient’s observations and clinical signs to imitate those seen in hypovolaemic patients [15].

### Tissue damage

Significant tissue damage has been proposed to cause a “pressor response” [16–18]. Increased sympathetic outflow results in tachycardia and increases in blood pressure mediated by vasoconstriction of the peripheral vasculature. It is thought that tissue damage also creates changes in the brainstem that alters the baroreceptor reflex, which allows sustained hypertension and tachycardia [17].

### Pain, afferent signals and autonomic networks

**Fig. 1:**
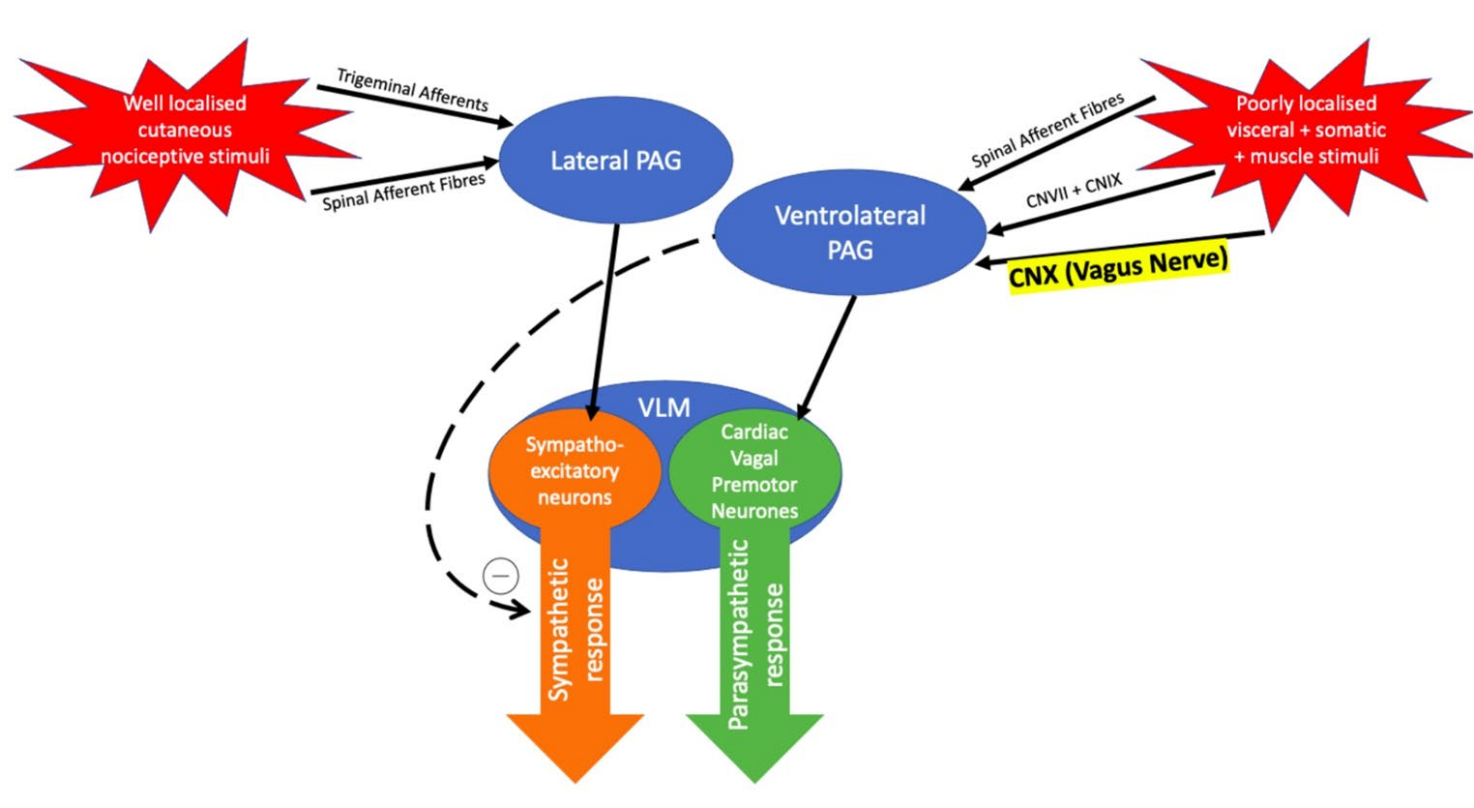
Central integration of peripheral stimuli by the periaqueductal grey (PAG) and ventrolateral medulla (VLM) causes a potentially varied response to injury. “CN” = Cranial Nerve

Pain is a complex phenomenon, involving a significant psychological component. In the context of trauma, it is caused by somatic or visceral lesions, or both [19]. Neuronal structures associated with the processing of painful stimuli are connected with centres of autonomic outflow to the heart. Evidence suggests that both the parasympathetic and sympathetic arms of the autonomic nervous system can be activated by pain [20]. Trigeminal and spinal afferents carry information regarding cutaneous nociceptive stimuli to the lateral periaqueductal grey (PAG) which initiates a sympathetic (hypertensive and tachycardic) response via the sympatho-excitatory neurones in the ventral lateral medulla (VLM). Information regarding non-specific somatic and visceral stimuli (such as visceral distension and stretch) is received by the ventrolateral PAG which projects fibres to the cardiac vagal premotor neurones in the VLM, as well as the medullary raphe which has an inhibitory effect on sympathetic outflow [21]. Together these neuronal centres cause a hypotensive and bradycardic response. Additionally, the emotional response to pain is mediated by the amygdala and its many connections to other centres of autonomic output [20]. There is evidence that the dominating output (sympathetic or parasympathetic) may depend on the characteristics of the patient [22], their affective state and the type and severity of stimulus [23]. Figure 1 illustrates the pathways thought to be responsible for the autonomic consequences of different stimuli.

### Vagally mediated reflex syncope

Reflex syncope is a phenomenon whereby patients develop profound bradycardia and hypotension [24], resulting in inadequate cerebral blood flow. It is thought to result from withdrawal of sympathetic tone (“vasodepression”) and stimulation of the vagus nerve (“cardioinhibition”) [24]. The response is associated with a variety of stimuli, including pain, venous pooling and emotional stimulation. Brignole et al. [25]. classify reflex syncope into four main categories:

1. Vasovagal (stimuli include fear, pain and instrumentation).
2. Situational (stimuli include gastrointestinal stimulation and exercise).
3. Carotid sinus syndrome.
4. Non-classical forms.

Despite the various terminology, all categories are mediated by the vagus nerve which is responsible for slowing the heart rate. These responses are also associated with an autonomic prodrome consisting of pallor, sweating and nausea [25].

### Integration

**Fig. 2:**
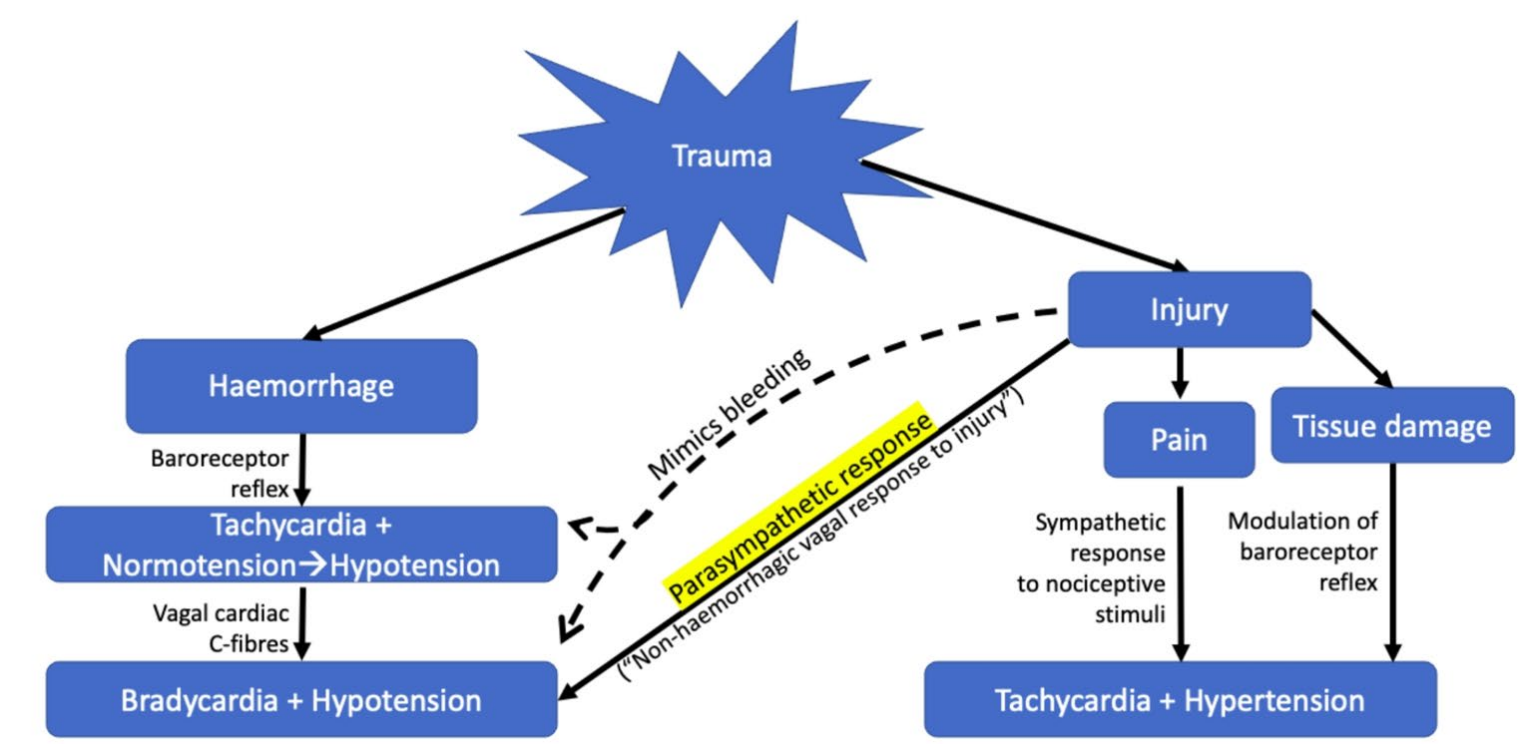
A parasympathetic response to injury may cause a patient to become hypotensive and bradycardic, thus mimicking a patient who is experiencing hypotension and bradycardia due to inadequate filling on the heart

Trauma can cause diverse physiological consequences. However, a patient’s heart rate and systolic blood pressure for example, are only linear variables. Interpreting these vital signs in the context of trauma requires knowledge of the underlying and dominant autonomic processes. The diagram below summarises the possible pathways that result in raised or lowered heart rates and blood pressures following trauma (Fig. 2). A trauma patient may be experiencing bradycardia and hypotension for different reasons: haemorrhage, or a parasympathetic response to injury which may mimic bleeding. This review will not discuss the cardiovascular consequences of impact brain apnoea [26, 27] and spinal cord damage [28]. These causes of hypotensive bradycardia following trauma are believed to be caused by different pathophysiology.

### “Vagal”

The term “vagal” only describes that relating to the vagus nerve. Yet, the vagus nerve is involved in many aspects: general visceral afferent fibres; afferent C-fibres in the left ventricular myocardium; and efferent fibres that innervate the sinoatrial node to control heart rate. According to a survey of UK-based prehospital clinicians undertaken by the authors, there is no consensus on whether, in the context of trauma, “vagal” refers to a pre-terminal hypovolaemic state with vagally mediated bradycardia, and/or a transient response to noxious stimuli with no significant hypovolaemia (Supplementary file 1).

The authors suggest that the term “haemorrhagic vagal response” should describe hypotensive bradycardia due to exsanguination, and “non-haemorrhagic vagal response to trauma” (NHVRT) should describe the vagal response to pain, visceral manipulation and emotional stimuli.

### Aims and rationale

An understanding of the pathology that is predominantly responsible for bradycardia and hypotension seen in trauma patients may inform management. Interventions such as blood transfusion, thoracotomy, and resuscitative endovascular balloon occlusion of the aorta carry a risk and should therefore not be initiated without consideration of non-haemorrhagic vagal responses to trauma. It is therefore important that the nature and causes of non-haemorrhagic responses to injury are understood so that these patients can be recognised and treated appropriately.

This review aims to summarise the patterns of injury, patient characteristics and clinical signs described in any primary literature that has previously documented non-haemorrhagic vagal responses to trauma. By doing so, this study will identify, if possible, any associations with such a parasympathetically predominant response in order to help clinicians assess the likely pathology behind bradycardia and hypotension following trauma.

## Review

### Methods

Four databases (PubMed, Embase, Scopus and Web of Science) were searched in December 2022 using expanded search terms of: Vagal response AND (cause OR patient characteristics). Patients sustaining injuries through both traumatic and iatrogenic mechanisms (surgical procedures) were included. The following inclusion and exclusion criteria were applied during screening of titles and abstracts (Table 1).

**Table 1:** Inclusion and exclusion criteria used in literature search to investigate the likelihood of a NHVRT

Inclusion criteria	Rationale
Humans	This review seeks to provide information to prehospital clinicians, who encounter adult and paediatric trauma
Adults and children	
English language	Constraints of time and available expertise
Primary data	This review aims to identify specific patterns of injury and patient characteristics, which requires primary data where possible
**Exclusion criteria**	**Rationale**
Hypotension and bradycardia attributable to confirmed blood loss	The aim of this study is to identify risk factors for non-haemorrhagic vagal response in the prehospital phase of treatment
Vagal response elicited deliberately or expected due to autonomic targeted intervention^1^	This study examines the response to trauma as opposed to planned interventions
Non-traumatic cause of vagal response	This literature does not relate to the prehospital cardiovascular resuscitation of trauma patients
Non-cardiovascular vagally mediated responses^2^	
Sub-clinical vagal responses^3^	
Studies in foetuses	
Cardiac syncope or cardiovascular responses of non-vagal origin (e.g. tachyarrhythmias)	
Diagnostic test studies	

^1^Examples include: vagal nerve stimulation for treatment of epilepsy, neuroablation surgery, pulmonary vein isolation, ganglionated plexi ablation, atrial ablation, studies that examine the efficacy of anaesthetic drugs or cardiac medication

^2^Examples include: gastrointestinal motility, cough and immunological changes

^3^Examples include: changes only seen in cortisol levels, pancreatic secretions and heart rate variability

Results were compiled. Duplicates, non-English language and retracted articles were removed. Records with titles and abstracts that met the exclusion criteria were removed before retrieving full text. Following full text review, articles that met the exclusion criteria were removed. Google Scholar and the bibliographies of included studies were searched to include any evidence not found during the initial literature search.

## Results

**Fig. 3:**
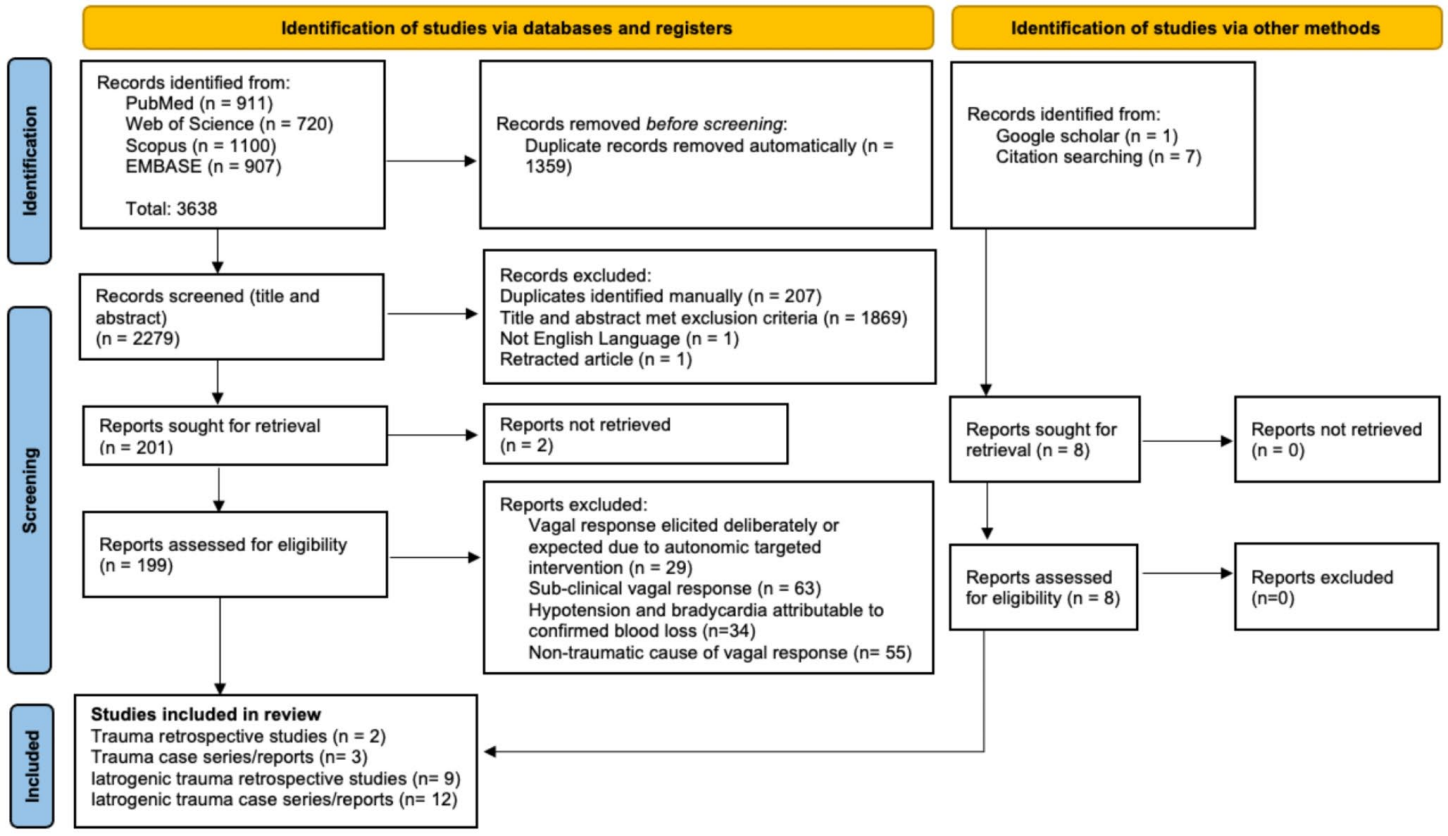
PRISMA diagram [29] displaying the identification of records relevant to the assessment of the likelihood of a non-haemorrhagic vagal response to trauma

Searching four databases returned a total of 3638 results, of which 1359 were automatically identified as duplicates. Initial screening removed a further 2078 results. The full text of two papers could not be retrieved and 199 papers were screened as full texts. Of these, 174 were removed because they met the exclusion criteria. Eight additional papers were identified from citation searching and Google Scholar (Fig. 3). Supplementary file 2 summarises the included studies.

### Patterns of injury

**Fig. 4:**
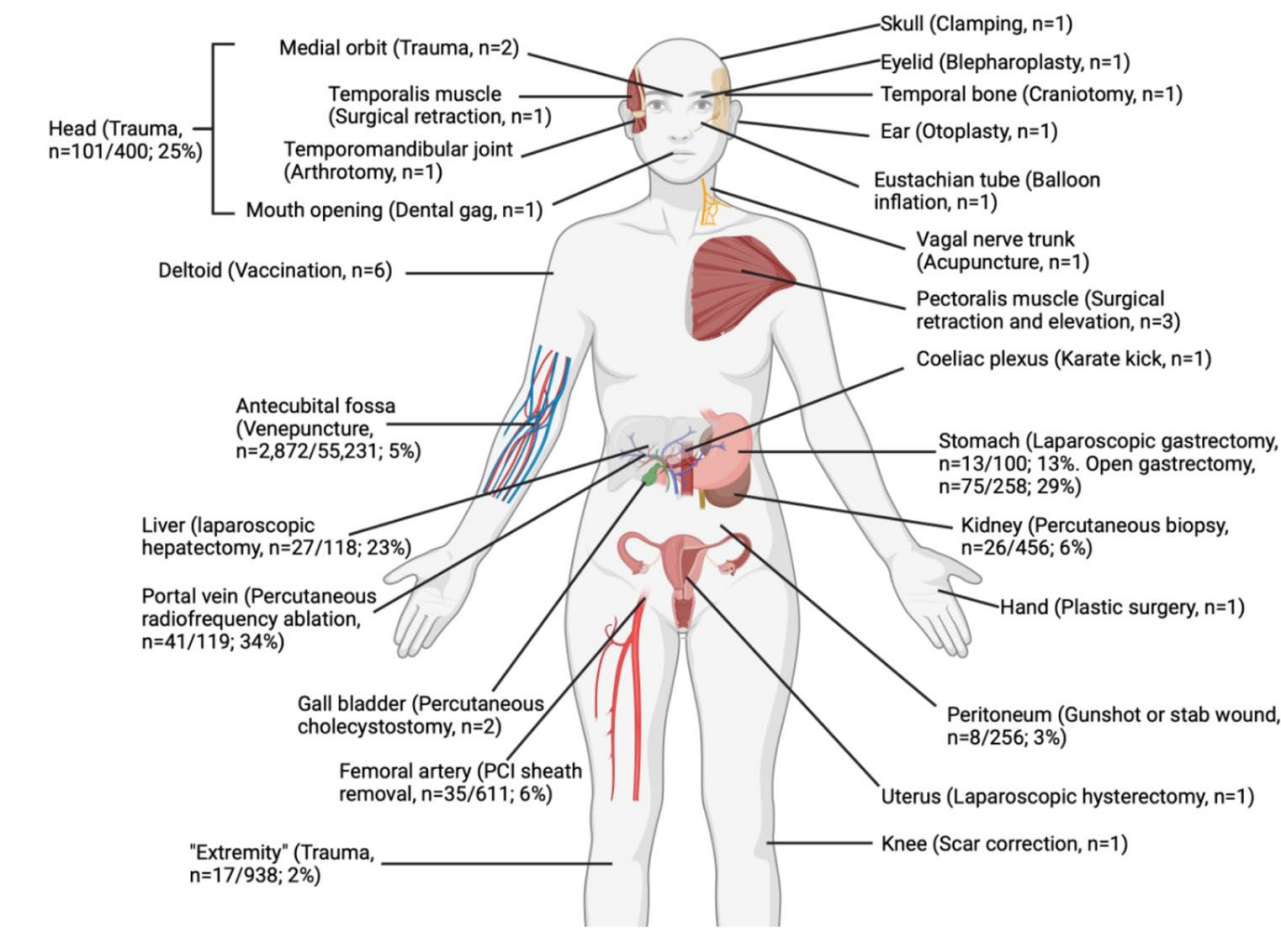
Anatomical locations of trauma and iatrogenic trauma that has elicited non-haemorrhagic vagal responses. Case reports and series are indicated by the number of patients reported. Analytic studies are indicated using the fraction and percentage of patients in whom a vagal response was recorded. Image created in BioRender

The anatomical locations of trauma and procedures that have elicited a non-haemorrhagic vagal response are summarised in Fig. 4.

### Demographics

Of the nine studies of iatrogenic trauma, four [30–33] showed a significant positive correlation between increasing age and a non-haemorrhagic vagal response. However, Takeuchi [34] and Juergens [35] were able to report no such link. Case reports of patients aged between 5 [36] and 74 [37] suggest that a non-haemorrhagic vagal response is possible in all ages. Female sex was related to an increased risk following hepatic radiofrequency ablation [31], and venepuncture [33, 38].

### Timeframes: onset

No trauma studies measured time from the point of injury when recording the progression of vital signs. Of the three included case reports, only one patient was reported to become symptomatic “immediately”, according to bystanders [39]. Surgical studies offer a more reliable account of the time interval between ”knife to skin” and onset of haemodynamic changes. Intervals ranged from “during blood withdrawal” [33], to 7.5 min [40], 13.1 min [30], and during the “early phase” of an operation [41]. Takese’s six cases of “delayed” reactions [42] include onset times up to 40 min. Bigongiari’s renal biopsy report described a reaction “one hour after the biopsy” [37].

### Timeframes: offset

Few studies commented on the time taken for a non-haemorrhagic reaction to resolve. Three surgical studies included protocols for administration of atropine or ephedrine following bradycardia, which obscures the natural timeframe of resolution [30, 31, 40]. However, in patients of one study where this was not administered, the majority of patients returned to normal haemodynamic parameters “within 30 s” [31]. Lee reported that patients experiencing hypotensive bradycardia following laparoscopic hepatectomy took between 12 and 408 s to recover their mean arterial pressure (MAP) above 65mmHg [40]. Similarly, case reports described periods of asystole for 10–20 s [36, 43, 44]. Case reports, although a lower level of evidence, provide more detail on whether the offending manoeuvre was reversed and if this expediated resolution of haemodynamic changes. One case report described bradycardia for a prolonged period due to the continuous entrapment of extra-ocular muscles [45]. Conversely, another reported that “cessation of retraction [of the temporalis muscle] resulted in immediate return of sinus rhythm” [46].

## Discussion

The recognition of NHVRT consists of two elements: the exclusion of haemorrhage; and identification of signs and symptoms associated with parasympathetic predominance.

### Exclusion of haemorrhage

Exclusion criteria in the literature search removed cases of confirmed haemorrhagic vagal responses to trauma. In cases where bradycardia was not attributed to haemorrhage, it was not always clear how bleeding was ruled out except for two case reports that used autopsy [39] and CT scan [45]. However, many of the surgical procedures would have been unlikely to cause significant haemorrhage and others may have allowed direct visualisation of the structures at risk of bleeding. Zyśko et al.’s retrospective study of trauma patients [47, 48] referred to potential blood loss (epistaxis, suturing of wounds) but did not explain exclusion of a haemorrhagic vagal response. Thompson’s retrospective study [48] did not measure blood loss. Instead, Thompson controlled for haemorrhage in the analysis of blood pressure and heart rate by using haematocrit, volume of crystalloid administered, respiratory rate and systolic blood pressure. However, these methods may be flawed due to lack of reliability of haematocrit in the hyperacute phase of exsanguination [49], inter-clinician variability of crystalloid administration and the use of blood pressure as both a control and dependant variable. Overall, none of the included studies ruled out haemorrhage using tools available to the prehospital clinician.

### Parasympathetic predominance

The manifestation of autonomic changes is measured in a variety of ways. Blood pressure and heart rate were used by 23 of the 26 included studies and case reports of NHVRT.

There is no consensus on the point at which haemodynamic parameters indicate a non-haemorrhagic vagal reaction to injury, although an absolute heart rate of below 60 bpm is commonly used [30, 32, 35, 40, 41] despite being physiological baseline for some patients [50]. Larger observations of normal populations have suggested the threshold for bradycardia under standard conditions be revised to 50bpm [51]. Similarly, what may be considered “normotension” in an otherwise hypertensive elderly patient may represent hypotension on an individual level [11]. Therefore, studies that use relative reductions in heart rate and blood pressure provide a better indicator of the changes to autonomic outflow that dictate these vital signs. However, no literature in the trauma population used this approach due to patient presentation after injury. Only in the surgical population could such a baseline be established. Furthermore, the use of recreational drugs (seen in over 10% of trauma patients in an Australian trauma population [52]) may affect blood pressure [11], which itself is not always measured accurately using non-invasive methods [53]. Therefore, the identification of other manifestations of autonomic disruption such as cerebration, diaphoresis and gastrointestinal symptoms may be considered.

Takeuchi [34] and Tomita [33] used assessments of weakness, sweating, loss of consciousness, nausea, vomiting and abdominal discomfort by healthcare professionals to assess the likelihood of a vagal reaction. Meanwhile, Zyśko [47] and Inaba [38] used self-reported measures of cerebral function (transient loss of consciousness, dizziness etc.) to diagnose a vagal reaction. Such self-reported outcomes are liable to responder bias due to the risk of traumatic brain injury [25].

### Patterns of injury

Many studies and case reports suggest that injury and bleeding into sites of autonomic significance may cause a vagal response. Autopsy reports of limited bleeding around the “upper vagal nerve trunk” [54] and celiac plexus [39] suggest that a non-haemorrhagic vagal response was elicited from the effect of blood compressing these autonomic structures. Park’s study [31] of hepatic radioablation suggested that patients’ heart rates were more likely to decrease if contact was made with the central portal vein, while the two cases presented by vanSonnenberg [55] describe a vagal reaction from drainage of, or contact with the gall-bladder. These structures are known to be densely innervated by vagal visceral afferent fibres in rats and dogs [56, 57].

Autonomic reflexes triggered such as the occulo-cardiac reflex [45, 58] and trigemino-cardiac reflex [36, 43, 59] were reported in a selection of cases reports. Yet, the included reports describe vagal reactions from a wide variety of peripheral structures^2^. Such stimulation may cause a parasympathetic response to pain due to complex emotional processes related to the perception of the stimulus [21]. It is also possible that the NHVRT in these patients arises from somatic inputs, which is known to be able to cause parasympathetic responses [60].

Only Thompson’s study [48] set out to investigate the hypothesis [61] that the site of injury and location of bleeding impacts the likelihood of a NHVRT. The study concluded that isolated penetrating abdominal trauma (*n* = 8) was not more likely to cause hypotensive bradycardia than isolated extremity trauma (*n* = 17). Although the validity of this study is undermined by the small number of patients and limitations in controlling for volume discussed earlier, this finding is in line with the many non-haemorrhagic vagal responses to peripheral trauma found by this review.

The magnitude of stimulation may also increase the likelihood of a NHVRT. Only surgical studies [30, 32] were reliably able to report the extent of injury or visceral manipulation. Kim’s study [30] found that an open procedure (associated with a greater degree of wall retraction and visceral manipulation) increased the risk of “reflex bradycardia” (OR = 3.184, *p* = 0.003). However, other variables associated with more extensive operations such as type of anaesthetic and patient comorbidities were not controlled, subjecting these findings to bias.

### Patient characteristics

The weak association between sex and a non-haemorrhagic vagal response, and variety of patient ages suggest that these patient characteristics may be poor indicators of the likelihood of a NHVRT.

Pre-operative bradycardia was identified as a risk factor by Kim [30] and included in the HEART score by Cheung [32]. However, both studies used an absolute threshold for bradycardia (< 60 bpm) and so it is unknown whether those with pre-operative bradycardia had a truly non-haemorrhagic vagal response to visceral manipulation. It has been suggested that athletes with a low resting heart rate have a greater degree of vagal tone [62]. However, this review found no evidence that a non-haemorrhagic vagal response to trauma is limited to athletes.

Pre-existing cardiovascular disease may represent a risk factor for developing hypotensive bradycardia in response to injury. Preoperative hypertension [40], revised cardiac risk index [32], angiotensin blocker medication use [32, 40] were identified as a risk factors by Lee and Cheung. The extent to which the anaesthetic is responsible for the haemodynamic changes in these co-morbid patients is unclear. Therefore, these findings may not be generalisable to trauma patients. Previous emotionally provoked vaso-vagal episodes were reported to increase the likelihood of a vagal reaction to head injury [47] and venepuncture [38]. This concurs with other studies [63] that suggest some individuals are more psychologically predisposed to vagal episodes than others. While comorbidities of a patient and previous episodes of vagally-mediated syncope may be associated with a non-hameorrhagic vagal response to trauma, this information may not be available to the clinician at the point of differentiating this pathology from bleeding.

### Timeframes

The included literature details a wide spectrum of timeframes over which hypotensive bradycardia develops and resolves. Onset varies between “immediate” [39] and up to 60 minutes [37]; resolution between 10–20 seconds [36, 43, 44] and 24 hours [45]. Furthermore, it remains unclear whether the non-haemorrhagic vagal response to injury requires continuous afferent signals from the injured area, or whether this is a reaction that can persist or even develop despite removal of the initial stimulus. It should be considered that there are a number of proposed mechanisms by which a non-haemorrhagic vagal response occurs, including visceral manipulation [64] and psychological stimulation [25]. Further research is required to investigate whether the stimulus that causes a non-haemorrhagic response dictates the timeframe of development and recovery. However, case reports of a NHVRT in patients under general anaesthetic (where visceral manipulation may be the likely trigger) and receiving injections (where fear may be considered a more likely trigger than visceral manipulation) suggest that a spectrum of onset and resolution times can occur in both groups.

### Limitations

This review found a small number of events in which hypotensive bradycardia following trauma could be attributed to the NHVRT. Only one paper [48] quantified the likelihood of NHVRT with different patterns of trauma. To expand the volume of available literature, “iatrogenic trauma” was considered which, although providing useful information regarding patterns of injury, patient characteristics and timeframe of symptom development, should be considered different to other forms of physical trauma for a number of reasons. Firstly, the non-haemorrhagic vagal response has a psychological element. Elective surgical patients rarely see the site of operation, whereas trauma patients may be immersed in an environment of fear and be able to see their own and others’ injuries. Secondly, surgical patients receive pain relief before incision. While this did not prevent a vagal response in all patients, the impact on nociceptive and visceral afferent fibres may affect the generalisability of the findings to trauma patients who do not receive analgesia or anaesthesia before their injuries. Thirdly, anaesthetic agents may have an impact on haemodynamic stability [65] and the drug history is known in the surgical population. Trauma patients, however, are under no such anaesthetic and may be intoxicated with haemodynamically destabilising recreational substances. Thus, the included literature is too heterogenous to meaningfully synthesise the events to estimate the true incidence of non-haemorrhagic vagal responses to injury and identify statistically significant risk factors.

## Conclusions

Clear terminology is needed to differentiate the cardiac vagal C-fibre mediated response to profound hypovolaemia from the parasympathetic response to injury without significant haemorrhage. This review has collated the patterns of injury, patient characteristics and clinical signs described in previous reports of non-haemorrhagic vagal responses to trauma. In summary, non-haemorrhagic vagal responses to trauma can be caused by a variety of injury patterns and occur in a diverse group of patients. The timeframe over which the response develops and resolves is also heterogenous. This makes recognition of a non-haemorrhagic cause of hypotensive bradycardia difficult using these parameters. Therefore, it remains a major clinical challenge to differentiate these patients and avoid unnecessary intervention in those that are not bleeding in the hyperacute phase. Since this review has demonstrated that it is not clinically possible to confidently diagnose or rule out a non-haemorrhagic vagal response to trauma, education of clinicians and awareness of this clinical conundrum remain paramount. A renewed focus on understanding the physiological response to haemorrhagic and non-haemorrhagic injury may reveal other clinical and biochemical changes specific to each pathology which may contribute to informing nuanced care. Furthermore, the emerging use of artificial intelligence to analyse changes multiple clinical parameters may assist future clinicians in their interpretation of vital signs in the hyperacute phase of injury.

## Electronic supplementary material

Below is the link to the electronic supplementary material.

Supplementary Material 1

Supplementary Material 2

## Data Availability

No datasets were generated or analysed during the current study.

## Abbreviations

ATLS: Advanced Trauma Life Support
CN: Cranial Nerve
HEART: Heart rate, Elderly age, preoperative renin-Angiotensin blockade, Revised cardiac risk index, and Type of surgery
NHVRT: Non-Haemorrhagic Vagal Response to Trauma
PAG: Periaqueductal grey
VLM: Ventral lateral medulla

## Footnotes

1 Defined in both studies as a heart rate (HR) below 90 beats per minute (bpm) and systolic blood pressure (SBP) below 90mmHg.

2 Hand and knee [41], antecubital fossa [33, 38],deltoid [42], femoral triangle [35], and pectoralis muscle [50].
