# Supplementary material for: The non-haemorrhagic vagal response to trauma: a review of hypotensive and bradycardic responses to injury in the absence of bleeding
Source: Eur J Trauma Emerg Surg. 2024 Sep 4;50(5):1995–2004. doi: 10.1007/s00068-024-02648-y (PMC11599317; doi:10.1007/s00068-024-02648-y)
Supplement: Supplementary file 1 — Supplementary Material 1 [file 68_2024_2648_MOESM1_ESM.docx]

**Clinician Questionnaire**

A questionnaire was created in order to characterise the approaches to the vagal response by prehospital clinicians (Figure S1).

Q1: Please confirm that you are a prehospital clinician who has experience assessing and

managing trauma patients

[Radio button]

- Yes

- No

Q2: What does the term “vagal” mean to you, in the context of patients who are hypotensive, bradycardic and may also be hypovolaemic due to haemorrhage?

[Tick box]

• A pre-terminal hypovolaemic state with vagally mediated bradycardia

• A transient response to noxious stimuli, with NO significant hypovolaemia

• Both of the above clinical scenarios

• Other (free text)

Q3: What factors might raise or lower your suspicion of a non-haemorrhagic vagal response to injury?

[Tick box]

• Pattern of injury unlikely to cause haemorrhage

• Mechanism of injury

• Degree of expected visceral manipulation

• Age, sex, or other demographic factors

• Co-morbidities of the patient

• Head or high spinal injury

• Other (free text)

Q4: In a bradycardic, hypotensive patient who may or may not be bleeding, how would you differentiate between hypovolaemia or a vagal, non-haemorrhagic, response to injury?

[Tick box]

• Timeframe of symptoms

• Response to fluid challenge

• Response to analgesia / sedation

• Other symptoms such as diaphoresis, pallor, air hunger etc.

• Other (free text)

Q5: Are you aware of the implications of the vagal reflex on the reliability of existing

haemorrhage assessment tools (Shock index; hateful 8; systolic blood pressure alone; ATLS classes of haemorrhage)?

[Radio buttons]

- Yes

- No

Q6: How do you approach the use of these scoring systems in patients who may be displaying a non-haemorrhagic vagal response?

[Free text]

**Figure S1:** Questionnaire

This was distributed online, with a consent form and participant information sheet, to 247 experienced, UK-based prehospital medicine clinicians using informal professional networks. Ethical approval was granted^[[1]](#footnote-1)^. Results were collected on March 1^st^ 2023. Free text responses were analysed using an inductive content analysis approach to capture and code concepts not included in multiple choice options in Questions 2-4.

**Question 1-2: results**

30 out of 247 experienced prehospital clinicians consented and responded to the questionnaire. 19 clinicians (64%) considered the “vagal” response to encompass both haemorrhagic and non-haemorrhagic trauma physiology (Figure S2).


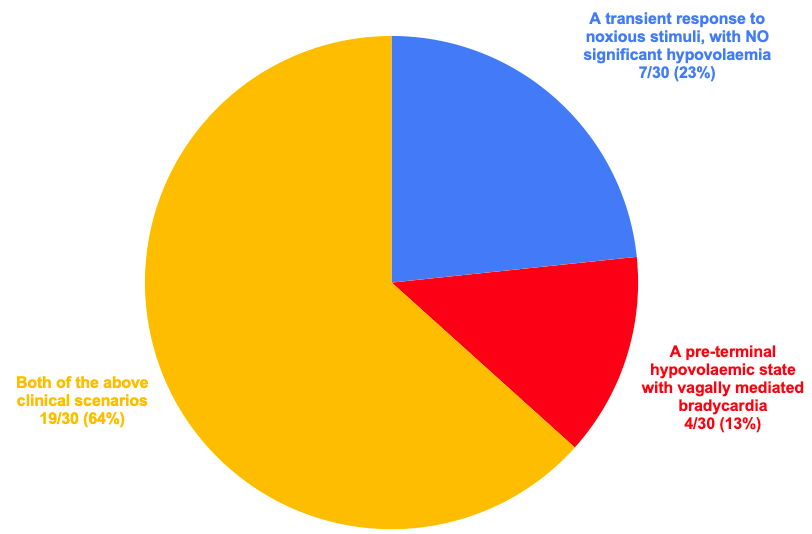


**Figure S2:** Responses to the question: “What does the term “vagal” mean to you, in the context of patients who are hypotensive, bradycardic and may also be hypovolaemic due to haemorrhage?”

1. Ethical approval was granted by The Institute of Health Sciences Education, Queen Mary University on 27^th^ January 2023. (Reference: IPREC230127.WOO). [↑](#footnote-ref-1)
