# Supplementary material for: The non-haemorrhagic vagal response to trauma: a review of hypotensive and bradycardic responses to injury in the absence of bleeding
Source: Eur J Trauma Emerg Surg. 2024 Sep 4;50(5):1995–2004. doi: 10.1007/s00068-024-02648-y (PMC11599317; doi:10.1007/s00068-024-02648-y)
Supplement: Supplementary file 2 — Supplementary Material 2 [file 68_2024_2648_MOESM2_ESM.docx]

# Summary of included literature

♂= % participants that are male; ⏱ = average age of participants

HR = Heart rate; bpm = beats per minute; SBP = systolic blood pressure; MAP = mean arterial pressure; VVR = vasovagal reaction

Table S1: Retrospective studies of vagal reactions to trauma in the absence of exsanguination

| **Author**  **Publication Year**  **Title**  **Type of Study** | **Population** | **Patient characteristics (sex, age, ethnicity, comorbidities)** | **Mechanisms of injury included** | **Time between injury and vagal reaction** | **How was vagal response measured?** | **Study findings** |
| --- | --- | --- | --- | --- | --- | --- |
| Zyśko et al., 2014 (48)  *History of syncope predicts loss of consciousness after head trauma: Retrospective study*  Retrospective study | 818 patients consecutive ED patients (319 reported previous traumatic head injuries) | ⏱ = 41  ♂= 56%  126 had history of vasovagal syncope. | High-energy trauma (high-speed road traffic accident either as pedestrian, cyclist or vehicle occupant, fall from a height, high-speed injury from a projectile or other object, beating, etc.)  or low-energy trauma (falling from upright position i.e. at ground level or low-speed injury from an object) | Not reported | Patient recalled TLOC following head trauma. Vagal reaction diagnosed according to “typical triggers and prodrome”. | Previous vasovagal syncope is associated with TLOC after head trauma (both high and low energy) independently of age, gender, and the mechanism of injury (OR 4.34, 95% CI 2.34–7.89, p < 0.001). |
| Thompson et al., 1990 (49)  *Relative bradycardia in patients with isolated penetrating abdominal trauma and isolated extremity trauma*  Retrospective study | 1194 patients presenting to hospital following trauma | Penetrating abdominal trauma:  ♂= 91.5%  ⏱ = 28.6  Isolated extremity trauma:  ♂= 82.8%  ⏱ = 33.9 | Penetrating abdominal trauma: 256  Isolated extremity trauma: 938 | Not reported | HR < 100  SBP < 100 | Isolated penetrating abdominal trauma: of 256 patients, 29 had a SBP below 100, of whom 8 showed relative bradycardia.  Isolated severe extremity trauma: of the 938 patients, 42 had a blood pressure below 100, of whom 17 showed relative bradycardia.  There we no significant correlations between injury pattern and incidence of relative bradycardia.  Every patient with relative bradycardia survived. |

Table S2: Case reports of vagal reactions to trauma in the absence of exsanguination

| **Author**  **Publication Year**  **Title**  **Type of Study** | **Patient characteristics (sex, age, ethnicity, comorbidities)** | **Mechanisms of injury included** | **Time between injury and vagal reaction** | **How was vagal response measured?** | **Findings** |
| --- | --- | --- | --- | --- | --- |
| De Froidmont et al. 2015 (35)  *Cardioinhibitory reflex due to a karate kick.* | 17 year-old male. Reportedly intoxicated. | Blunt trauma to abdomen (kick) | Immediate loss of consciousness according to bystanders. | Immediate unconsciousness. Complete asystole on arrival of medical team (10 mins from injury) | Cardioinhibitory reflex suggested as cause of death following post-mortem examination, which showed compression of coeliac plexus. |
| Hirjak et al. 1993 (58)  *Bradycardia after orbital injury – case report.* | 33 year old male.  No co-morbidities. | Penetrating injury to medial orbit | Not reported | HR = 30. BP = 75/40.  No significant blood loss. | Patients with orbital trauma should be observed due to this complication. Cardiovascular parameters normalise immediately following withdrawal of stimulus. |
| Despain et al. 2020 (41)  *Oculocardiac Reflex in a Pediatric Trauma Patient.* | 6 year-old male.  No co-morbidities | RCT causing facial trauma | Not reported | HR = 67; BP = 115/72 on scene.  HR = 61; BP = 121/72 during hospital assessment.  No significant blood loss. | Bradycardia resolved within a day as extraocular muscle entrapment improved |

Table S3: Studies of risk factors that predict vagal response to “iatrogenic trauma” in the absence of exsanguination

| **Author**  **Publication Year**  **Title**  **Type of Study** | **Population** | **Patient characteristics (sex, age, ethnicity, comorbidities)** | **Mechanisms of injury included** | **Time between injury and vagal reaction** | **How was vagal response measured?** | **Study findings** |
| --- | --- | --- | --- | --- | --- | --- |
| Kim et al. 2015 **(26)**  *Analysis of factors related to vagally mediated reflex bradycardia during gastrectomy*  Prospective observational study | 358 adult patients (age 18–70 years) | ♂=65%  ⏱=56.8  All patients indicated for surgery due to gastric cancer diagnosis | Laparoscopic gastrectomy  -or-  Open gastrectomy | 13.1 minutes from surgical incision | HR <50 bpm  -or-  HR = 50–59 bpm + SBP <70mmHg, associated with a “abdominal wall retraction, manipulation of abdominal visceral organs, or CO2 gas insufflation.” | Incidence of vagal response = 24.6 %  Factors independently associated with reflex bradycardia:   - - - Advanced age     - Preoperative bradycardia     - Open gastrectomy - “increased visceral manipulation and mechanical force from wound retractors”. (OR 3.184; 95 % CI 1.490–6.800; *p* = 0.003) |
| Park et al. 2009 **(27)**  *Prospective analysis of the pattern and risk for severe vital sign changes during percutaneous radiofrequency ablation of the liver under opioid analgesia*  Prospective observational study | 102 patients  (age 35 –85 years) | ♂=72%  ⏱=58.1  All patients indicated for surgery due to malignant tumours | Percutaneous hepatic radio-frequency ablation (RFA) | Not reported  Mean ablation time = 13.6 minutes | SBP or HR reduction > 30% | Incidence of vagal response = 35%  Factors independently associated with cardioinhibitory response:   - - - Advanced age     - Female sex     - Contact with the central portal vein |
| Lee et al. 2020 **(36)**  *Symptomatic (Hypotensive) Bradycardia During Laparoscopic Living Donor Hepatectomy: Incidence and Risk Factors*  Retrospective study | 118 healthy donors | ♂=58%  ⏱ =32.5 | Laparoscopic living donor [hepatectomy](https://www.sciencedirect.com/topics/medicine-and-dentistry/hepatectomy) (including CO_2_ insufflation to 12mmHg) | 7.5 minutes | HR < 60bpm  MAP < 65mmHg | Incidence of vagal response = 23%  Factors independently associated with hypotensive bradycardia:   - - - Preoperative hypertension     - Angiotensin receptor blocker medication |
| Cheung et al. 2015 **(28)**  *Predictors of intraoperative hypotension and bradycardia*  Prospective observational study | 193 patients elective surgery patients | ♂=34%  ⏱=67.6 | Elective, non-cardiac surgery.  59.1% underwent major surgery | Not reported | SBP <90 mmHg for >5 minutes  -or-  MAP reduction > 35%  -or-  <60 beats/min for >5 minutes. | Incidence of intraoperative hypotension or bradycardia = 66%  HEART score predicted intraoperative hypotension or bradycardia (OR = 2.51; 95% CI = 1.79-3.53)   - - - Heart rate (<60 beats/min)     - Preoperative hypotension (<110/60 mm Hg)     - Elderly age (>65 years)     - Preoperative renin-Angiotensin blockade (angiotensin-converting enzyme inhibitors, angiotensin receptor blockers, or beta-blockers)     - Revised cardiac risk index (3 points)     - Type of surgery (major surgery) |
| Takeuchi et al. 2018 **(30)**  *Manual compression and reflex syncope in native renal biopsy*  Retrospective study | 456 patients indicated for renal biopsy | ♂=62%  ⏱=65.7 | 3 core biopsies form inferior pole (16-guage needle) under local anaesthetic and ultrasound guidance | Not reported  Compress-ion group received manual compress-ion for 15 minutes | Drop in SBP > 20mmHg + nausea, vomiting, abdominal discomfort, or excessive perspiration. | Incidence of transient hypotension = 6%  Compression increased risk of transient hypotension (Adjusted OR=3.27; 95% CI=1.36-7.82)  No patient characteristics (age, sex, BMI, resting SBP) correlated with transient hypotension. |

| Juergens et al. 2008 **(31)**  *Vaso-vagal reactions during femoral arterial sheath removal after percutaneous coronary intervention and impact on cardiac events*  Prospective observational study | 611 PCI patients with a femoral sheath | ♂=74%  ⏱=60.1 | Sheath removal using manual compression | Not reported | HR < 60bom  SBP < 100mmHg (or >10% below baseline) | Incidence of vagal reaction = 5.7%  Only larger sheath size was associated with vaso-vagal reaction |
| --- | --- | --- | --- | --- | --- | --- |
| Hurwitz & Ogilvie. 1995 **(37)**  *Reflex bradycardia in out-patient surgery done under local anesthesia*  Retrospective study | 432 consecutive plastic surgical patients operated under local anaesthesia | ♂=Not reported  ⏱=38 | Out-patient plastic surgery (variety of sites and indications) | Not reported  “Early phase of operation” (8/12) or “during injection of local anaesthetic (4/12) | Heart rate below 60bpm (range 35-56) | Incidence of reflex bradycardia = 2.8%  Operations that caused reflex bradycardia included:   - - - Facial surgery (including blepharoplasty)     - Otoplasty     - Hand surgery     - Scar correction (knee)   No statistical analysis of associated risk factors |
| Inaba et al. 2013 **(34)**  *Analysis of a questionnaire on adverse reactions to blood donation in Japan*  Observational study | 55,231 volunteer blood donors | ♂=71%  ⏱=41.2 | Venepuncture (Donation of 400ml of blood) | Not reported | Self reported: The major adverse reactions were vertigo or dizziness (66.5%), nausea (12.4%), and fainting or stumbling (3.0%). | Incidence of adverse reaction = 5.2%    Factors associated with increased risk of adverse reaction:   - - - History of vasovagal response     - Female sex (especially with low body weight)     - First- or second-time donors     - Low haemoglobin concentration     - Phobia of the sight of blood. |

| Tomita et al. 2002 **(29)**  *Vasovagal reactions in apheresis donors*  Observational study | 28189 whole blood donors and 21245 apheresis donors | ♂=70%  ⏱=Not reported | Venepuncture  Whole blood donation (200-400mL) and apheresis (combined plasma or platelet extraction (volume not stated) | Not reported  “During or after blood with-drawal” | Identification of VVR by experienced nurses according to “weakness, pallor, nausea, sweating, and fainting” | Incidence of VVR in apheresis donors was 0.99% in men and 4.17% in women (no statistical analysis)  Incidence of VVR in whole blood donors decreased with age (no statistical analysis) |
| --- | --- | --- | --- | --- | --- | --- |

Table S4: Case reports and case series of vagal reactions to “iatrogenic trauma” in the absence of exsanguination

| **Author**  **Publication Year**  **Title**  **Type of Study** | **Patient characteristics (sex, age, ethnicity, comorbidities)** | **Mechanism of injury** | **Time between injury and vagal reaction** | **How was vagal response measured?** |
| --- | --- | --- | --- | --- |
| Takase et al. 2022 **(38)**  *Delayed Vasovagal Reaction with Reflex Syncope Following COVID-19 Vaccination*  Case series (6 patients) | Whole cohort not reported.  Of the 6 patients experiencing delayed reaction:  ♂=66%  ⏱=23.2 | COVID-19 vaccination | Average = 23 mins  (Range = 10-40mins) | HR < 40bpm for >20 secs +  SBP < 90mmHg |
| vanSonnenberg et al. 1984 (43)  *Life-threatening vagal reactions associated with percutaneous cholecystostomy*  Case series (2 patients) | 64 year old man, with temp 39.4 and right upper quadrant pain and a distended gall bladder on ultrasound scan. | Percutaneous insertion of pigtail catheter into gallbladder and drainage of 300mL bile. | Immediately after drainage of bile | Heart rate initially dropped to 40bpm, then patient suffered a cardiac arrest. |
|  | 74 year old man with right upper quadrant pain and temp of 37.2-38.6. History of renal failure and pericardial effusion. | Percutaneous insertion of pigtail catheter into gallbladder. “Attempts of aspiration [>25mL] or catheter manipulation against gallbladder wall resulted in [vagal response].” | Immediately after aspiration or contact with gallbladder wall | HR dropped from 120 to 60bpm  SBP dropped from 100/60 to 65/0mmHg |
| Bigongiari et al. 1979 (33)  *Vagal hypotension after percutaneous biopsy: Possible confusion with hypovolemic shock*  Case series (2 patients) | 26 year-old woman, with a history of transient microscopic haematuria during pregnancy | Administration of intravenous contrast medium and percutaneous renal biopsy | 60 mins | HR = 63  BP = 70/40  Pt felt faint, nauseous, and turned pale |
|  | 70 year-old female with urinary retention | Administration of intravenous contrast medium and renal cyst puncture (including 4 unsuccessful attempts) | “Shortly after” | HR = 62  BP = 100/60  Stable haemoglobin and haematocrit |
| Schusterman & Schusterman. 2011 (44)  *Asystole in Young Athletic Women During Breast Augmentation: A Report of Three Cases*  Case series (3 patients) | Three healthy, adult female patients with a history of endurance training and resting bradycardia (heart rate = 60 beats per minute) | Subpectoral breast augmentation using inframammary access under general anaesthesia  Raising head of bed (x1)  Retraction-elevation of the pectoralis muscle (x2) | Immediate | Profound hypotension and transient asystole |
| Miyoshi et al. 2014 (40)  *Asystole following skull clamp to Chiari malformation*  Case report | A 17-year-old woman with Arnold–Chiari malformation type I and worsening syringomyelia | General anaesthetic and fixation of the head with a skull clamp | 3 seconds after clamping | 10 seconds of asystole, then spontaneous return of heart rate to 30bpm |
| Morey & Bjoraker. 1996 (39)  *Asystole during temporomandibular joint arthrotomy*  Case report | 41 year old woman.  No co-morbitidies other than oobesity. Previously successful general anaesthetics.  Only taking ibuprofen for preauricular pain.  Baseline BP = 125/50 and HR = 72 | TMJ arthrotomy, specifically irrigation of joint with water (20 degrees Celsius) | Immediate | Complete sinus arrest with recovery to 20 bpm after 20 seconds. |
| Chung et al. 2022 (45)  *Transient asystole during balloon dilation of the Eustachian tube: A case report.* | 33 year old woman.  Baseline BP 113/72 mmHg and HR=79bpm | Left tympanoplasty and left balloon dilation (12ATM) of the Eustachian tube | Immediate | Asystole |
| Imai et al. 2022 (46)  *Robotic-assisted laparoscopic hysterectomy and vasovagal reflex: A case report* | 46-year-old woman.  No comorbidities. Smoker. | Robotic-assisted laparoscopic hysterectomy, specifically manipulation of the uterine body. | Immediate | HR dropped from 60 to 30 bpm |
| Kariya et al. 1999 (47)  *Coronary artery spasm induced by trigeminal nerve stimulation and vagal reflex during intracranial operation*  Case report | 58 year old man with right facial pain. Mass in right petrosal apex. No other medical history. ECG showed a left bundle branch block.  Baseline BP = 138/62 and HR = 68 | Right temporal craniotomy. Specifically drilling of petrosal bone between mandibular and petrosal nerve.  General anaesthesia | 5 minutes | BP dropped from 120/60 to 75/40 and HR from 65 to 50. Ventricular tachycardia and then ventricular fibrillation occurred. |
| Hoshijima et al. 2021 (32)  *Asystole Triggered by the Mouth Opening With a Dental Mouth Gag Under General Anesthesia During Pediatric Oral Surgery: Report of a Rare Case*  Case report | 5-year-old boy. No co-morbidities. | Mouth opened with dental gag for extraction of maxillary supernumerary teeth | Immeditate | Asystole for 20.  When mouth opened more than 40cm, HR dropped from 100 to 70 bpm |
| Shakil et al. 2018 (42)  *Trigemino-cardiac reflex: A case report of intra-operative asystole in response to manipulation of the temporalis muscle*  Case report | A 60 year-old female. Co-morbidities not recorded | Retraction with moderate force of the temporalis muscle | Immediate | Asystole |
| Watanabe et al. 2015 (54)  *An autopsy case of vagus nerve stimulation following acupuncture*  Case report | 40-50-year-old-male. Co-morbidities not recorded | Needle acupuncture to the right cervical region  “Histopathology revealed severe  haemorrhaging in the upper vagal nerve trunk” | “Soon after” | Complained of nausea and lost consciousness. Cardiac arrest confirmed on ambulance arrival. |
